# Chemical Composition and In Vitro Antidiabetic Effect of Extracts from Ripe, Unripe, and Fermented Unripe *Cornus mas* L. Fruits

**DOI:** 10.3390/molecules30234625

**Published:** 2025-12-02

**Authors:** Karolina Bernacka, Agata Czyżowska, Małgorzata Małodobra-Mazur, Monika Ołdakowska, Anna Otlewska, Tomasz Sozański, Alicja Z. Kucharska

**Affiliations:** 1 Department of Fruit, Vegetable and Plant Nutraceutical Technology, Wrocław University of Environmental and Life Sciences, Chełmońskiego 37, 51-630 Wrocław, Poland; 2 Institute of Fermentation Technology and Microbiology, Łódź University of Technology, Wólczańska 171/173, 90-530 Łódź, Poland; agata.czyzowska@p.lodz.pl (A.C.); anna.otlewska@p.lodz.pl (A.O.); 3 Department of Forensic Medicine, Division of Molecular Techniques, Wrocław Medical University, M. Skłodowskiej-Curie 52, 50-369 Wrocław, Polandmonika.oldakowska@umw.edu.pl (M.O.); 4 Department of Preclinical Sciences, Pharmacology and Medical Diagnostics, Faculty of Medicine, Wrocław University of Science and Technology, Wybrzeże Wyspiańskiego 27, 50-370 Wrocław, Poland

**Keywords:** *Cornus mas*, cornelian cherry, plant extracts, chemical composition, iridoids, hydrolyzable tannins, antioxidant, insulin resistance, adipocytes

## Abstract

This study aimed to investigate the chemical composition, antioxidant activity, and in vitro antidiabetic properties of extracts obtained from ripe, unripe, and fermented (unripe) cornelian cherry (*Cornus mas* L.) fruits. Polyphenols were identified using UPLC-ESI-qTOF-MS/MS and quantified by HPLC-PDA. Antioxidant activity was evaluated using ABTS, DPPH, and FRAP assays, while enzyme inhibitory activity was determined for *α*-glucosidase and *α*-amylase. Additionally, the effects of *C. mas* extracts on insulin sensitivity in adipocytes were investigated. The study’s results showed that each of the extracts tested contained varying proportions of substances with proven health-promoting properties. The extract from ripe fruits was characterized by the highest loganic acid content, whereas the extract from fermented unripe fruits contained a high amount of gallic acid, released through the hydrolysis of tannins during fermentation. The extract from unripe fruits exhibited the highest tannin content and the strongest antioxidant activity. All extracts inhibited *α*-glucosidase and *α*-amylase to a similar extent and improved insulin-stimulated glucose uptake in 3T3-L1 adipocytes without affecting *INSR* or *SLC2A4* expression. In conclusion, extracts from unripe and fermented *C. mas* fruits may represent promising agents for alleviating insulin resistance and preventing type 2 diabetes.

## 1. Introduction

Insulin resistance (IR) is defined as a state in which glucose uptake in insulin-target tissues is disrupted, along with an impaired hepatic glucose output. Resistance of the tissues to insulin leads to increased blood glucose levels, hyperinsulinemia, *β*-cell dysfunction, and, finally, the development of type 2 diabetes mellitus (T2DM) [1,2]. In 2021, diabetes affected 10.5% of the world’s population aged 20–79 years. The percentage of people with diabetes is expected to increase to 12.2% by 2045. In addition, the percentage of people suffering from impaired glucose tolerance is expected to rise from 10.6% in 2021 to 11.4% in 2045 [3].

The first step in the treatment of IR and T2DM involves lifestyle changes. Physical activity, weight loss, alternative dietary patterns (low-fat, low-carbohydrate, Mediterranean-style diets), and chrononutrition may be beneficial for regulating postprandial glucose levels and alleviating IR [4]. The second step involves the use of insulin-sensitizing antidiabetic drugs (metformin and thiazolidinediones), *α*-glucosidase inhibitors, dipeptidyl peptidase-4 inhibitors, and finally insulin [1].

Despite insulin therapy and the use of oral drugs, including *α*-glucosidase inhibitors that are commonly used to alleviate hyperglycemia and treat diabetes, natural compounds found in plants play a significant role in the prevention and treatment of T2DM. Phenolic compounds are of particular interest due to their lack of side effects and high bioavailability [5]. In particular, flavonoids and tannins play an important role in supporting diabetes treatment due to their ability to inhibit enzymes responsible for carbohydrate hydrolysis, *α*-glucosidase, and *α*-amylase [5]. Examples of flavonoids with strong inhibitory activity are rutin and quercetin. Rutin and quercetin inhibited *α*-amylase with IC_50_ values of 1.71 mg/mL and 0.67 mg/mL, respectively, compared with 14.6 ± 1.70 mg/mL for the reference substance, acarbose [6]. *α*-Glucosidase activity was also inhibited by rutin and quercetin, with IC_50_ values of 272 and 5.1 µg/mL, respectively, much lower than that of acarbose (465 ± 37 µg/mL) [7]. The inhibitory activity was also confirmed for the gallotannin 3,4,6-tri-*O*-galloyl-D-glucose, which inhibited *α*-amylase and *α*-glucosidase with IC_50_ values of 335 ± 8.9 μM and 46.5 ± 1.6 μM, respectively [8].

Dietary sources of polyphenols include plant-based products, including drinks (tea and coffee), nuts (peanuts and pistachios), vegetables (onion and green leafy vegetables), and fruits (grapes, cherries, and pears), including the not well-known cornelian cherry fruit [9]. *Cornus mas* L. (cornelian cherry) is a shrub native to southern Europe and Western Asia. Ripe fruits are generally red, but cultivars with yellow, pink, and nearly black fruits are also harvested. Fruits are consumed fresh or used for the production of jam, juice, marmalade, compote, syrup, liquor, and wine [10,11]. Cornelian cherry fruits contain phenolic compounds, mainly flavonoids (anthocyanins and flavonols), phenolic acids, and hydrolyzable tannins. Iridoids are a characteristic group of secondary metabolites that are not widely distributed in the edible parts of plants. The predominant and characteristic iridoid of *C. mas* fruits is loganic acid, followed by cornuside, sweroside, and loganin, which are present in lesser amounts [11,12,13].

Previous research has indicated that unripe fruits may be of interest because of their varied chemical composition [14,15,16,17,18]. In contrast to ripe *C. mas* fruits, unripe (green) fruits do not contain anthocyanins but have significantly higher amounts of phenolic acids, flavanols, hydrolyzable tannins, and iridoids. The total amount of secondary metabolites decreased by approximately 4.9-fold during ripening from green to dark red [16]. Although unripe cornelian cherry fruits are not edible, they can be used as raw material for pickles or dietary supplements. In southeastern Poland, unripe cornelian cherry fruits are lactofermented in brine with condiments such as savory and bay leaves. The product, due to its form and taste, is called a ‘Polish olive’ and was historically a table olive substitute [19]. During fermentation, the chemical composition of fruits changes due to the activity of microbial enzymes. The biotransformation of phenolic compounds present in the raw material generally results in increased total phenolic content (TPC), antioxidant activity, and enhanced bioavailability of phenolics [20]. As information about the chemical composition and health-promoting potential of unripe lactofermented *C. mas* fruits is limited, the present study aimed to investigate the profile of biologically active compounds as well as the antioxidant and antidiabetic activities of fermented cornelian cherry.

In our previous study [21], we demonstrated that resin-purified extracts from the fruits of two *C. mas* cultivars may enhance glucose uptake and upregulate the expression of *Pparg* in insulin-resistant murine adipocytes. In the present work, we aimed to investigate the effects of extracts from ripe, unripe, and lactofermented *C. mas* fruits on insulin sensitivity and on the expression of key genes and transcription factors involved in the insulin signaling pathway. Furthermore, we compared the chemical composition and antioxidant properties of extracts derived from unripe, ripe, and fermented *C. mas* fruits. To the best of our knowledge, this is the first study to evaluate the *α*-glucosidase and *α*-amylase inhibitory activities and antidiabetic potential of extracts from ripe, unripe, and fermented fruits in a mouse preadipocyte cell model. In this study, we provide a comparative analysis of the chemical composition and biological properties of ripe, unripe, and lactofermented unripe *C. mas* fruits from the same cultivar for the first time. Moreover, in the present study, we prepared different product variants to determine the optimal fermentation conditions for producing fermented cornelian cherry.

## 2. Results and Discussion

The resin-purified extracts from ripe (fully matured), unripe (green), and unripe lactofermented fruits of *C. mas* were analyzed in terms of chemical composition, TPC, antioxidant activity, and insulin sensitivity in murine adipocytes.

### 2.1. Fermentation of Unripe Cornelian Cherry Fruits

Five products were obtained from the fermented unripe fruits before preparing the extract. These variants differed in the type of condiments used, addition of prebiotics (inulin), and fermentation method (spontaneous or starter-culture-induced). The starter strain used to prepare the products was *Levilactobacillus brevis* ZP HE1. The microbiota present in fermented cornelian cherry brines and microbial by-products are summarized in Table 1 and Table 2, respectively.

The total mesophilic count in the analyzed products ranged from 4.81 ± 0.56 to 6.43 ± 0.09 log CFU/mL. The highest yeast count was observed in product 4 (6.41 ± 0.74 log CFU/mL), whereas the lowest was detected in product 2 (3.08 ± 0.36 log CFU/mL). In the experiment conducted by Czyżowska et al. [22], yeast and mold counts in fermented, unripe *C. mas* fruits—both spontaneously and with lactic acid bacteria (LAB) starter cultures or black olive microbiota—were also high and ranged between 4.64 ± 0.15 and 5.96 ± 0.06 log CFU/mL. Members of the family *Enterobacteriaceae* were detected only in products 1 and 2, whereas LAB were present exclusively in products fermented with the addition of a starter culture (Table 1). Brine from product 5 was characterized by the highest content of lactic acid (519 ± 9.06 mg/100 mL) and acetic acid (134 ± 2.00 mg/100 mL). Although LAB were not detected in spontaneously fermented products, lactic acid was present at 78.5 ± 2.03 to 155 ± 2.11 mg/100 mL. This suggests that LAB were active during the early stages of fermentation but declined before the microbial analysis, which was performed after three months (Table 2). A reduction in the LAB population during the late fermentation phase has been observed in other lactofermented products, such as kimchi [23] and capers [24]. The content of propionic acid in all products ranged from 84.4 ± 2.60 to 295 ± 1.00 mg/100 mL.

Due to the absence of *Enterobacteriaceae*, high LAB count, and elevated concentrations of lactic and acetic acids, product 5 was considered to have undergone the most desirable fermentation process and was therefore selected for the preparation of the resin-purified extract and further analysis.

### 2.2. Identification of Phenolic Compounds and Iridoids in C. mas Extracts

The results of the qualitative analysis of the chemical composition of the fruit extracts are presented in Table 3. The compounds were identified based on their retention times and elution order in LC-MS, ion formulas of deprotonated molecules [M + H]^+^ or [M − H]^−^, and their fragment ions, as well as by comparison with data available in the literature. Anthocyanins were identified in the positive ion mode, whereas phenolic acids, hydrolyzable tannins, flavonols, and iridoids were identified in the negative ion mode. Iridoids and phenolic compounds, including phenolic acids, flavanols, and hydrolyzable tannins, were identified in each extract. The structural formulas of selected chemical compounds, along with selected chromatograms (UPLC-ESI-qTOF-MS/MS and UPLC-PDA), are provided in Figures S1–S8.

Four anthocyanin monoglycosides (compounds **1**–**4**) were identified in the ripe fruit extract: cyanidin 3-*O*-galactoside at *m*/*z* 449, cyanidin 3-*O*-robinobioside at *m*/*z* 595, pelargonidin 3-*O*-galactoside at *m*/*z* 433, and pelargonidin 3-*O*-robinobioside at *m*/*z* 595. All the mentioned anthocyanin glycosides were also identified by Dzydzan et al. [34] in *C. mas* ‘Podolski’ and by Klymenko et al. [11] in three Ukrainian *C. mas* cultivars (‘Ekxotychnyi’, ‘Kolarovyi Marka’, and ‘Uholok’), two *C. mas* × *C. officinalis* hybrids, and two *C. officinalis* cultivars. Cyanidin 3-*O*-galactoside and pelargonidin derivatives were also reported in *C. mas* fruits by Świerczewska et al. [33], although the authors did not provide the name of the cultivar.

Compounds **6**, **11**, **13**, **27**, and **46** were classified as phenolic acids and were detected in all analyzed extracts. Compound **6**, which produced a pseudomolecular ion at *m*/*z* 169, was identified as gallic acid, whereas compound 46, with a pseudomolecular ion at *m*/*z* 301 and fragment ions at *m*/*z* 275 and 249, was identified as ellagic acid, a dilactone of gallic acid didepside. Compounds **13** and **27** produced a fragment ion at *m*/*z* 163, indicating that they are *p*-coumaric acid derivatives, including coutaric acid. Compound **11** was identified as caftaric acid based on the fragment ions at *m*/*z* 149 and 179, which correspond to tartaric and caffeic acids, respectively. Coutaric and caftaric acids have been previously reported in an extract obtained from the red *C. mas* cultivar ‘Podolski’ and in an extract obtained from a mixture of two yellow *C. mas* cultivars, ‘Yantarnyi’ and ‘Flava’ [26]. Coutaric, caftaric, and other hydroxycinnamic acids are also characteristic of grapes, *Vitis amurensis* Rupr. (both wild and cultivated) [36] and *Vitis vinifera* L. (both ripe and unripe) [37,38].

The extract of ripe *C. mas* fruits contained six flavonol glycosides (compounds **43**, **44**, **47**, **50**, **51**, and **52**). The extract from unripe fruits contained all flavonol glycosides except compound **51** (kaempferol 3-*O*-rutinoside), whereas the extract from unripe fermented fruits contained only one flavonol glycoside, compound **47** (quercetin 3-*O*-glucuronide). Compounds **43**, **44**, and **47** produced a fragment ion at *m*/*z* 301, corresponding to quercetin, whereas compounds **50**–**52** produced a fragment ion at *m*/*z* 285, corresponding to kaempferol. Neutral losses of 308, 162, and 176 Da were assigned to rutinoside, hexoside (galactoside or glucoside), and glucuronide, respectively. Both compounds **50** and **52** give a pseudomolecular ion at *m*/*z* 447 and a fragment ion at *m*/*z* 285 and were identified as kaempferol hexosides [32]. According to Pawlowska et al. [32], these compounds were identified as kaempferol 3-*O*-glucoside (**50**) and kaempferol 3-*O*-galactoside (**52**). All the detected flavonol glycosides have been previously reported in *C. mas* fruits [32,33,34].

Compounds **5**, **7**, **20**, **42**, and **45** were identified as gallotannins because they produced fragment ions at *m*/*z* 169 and 125, which are characteristic of gallic acid residues. The simplest gallotannins present in all the extracts were mono-*O*-galloyl-*β*-D-glucose (**5**) and 7-*O*-galloyl-D-sedoheptulose (**7**). Mono-*O*-galloyl-D-glucose and galloyl-D-sedoheptulose have been previously reported in *C. officinalis* fruit extract [28], in *C. mas* fruit extract, and in ripe, fermented *C. mas* fruits processed using Symbiotic Culture of Bacteria and Yeast (SCOBY) [30]. More complex gallotannins included tri-*O*-galloyl-*β*-D-glucose (**20**) and two isomers of tetra-*O*-galloyl-*β*-D-glucose (**42**, **45**). Notably, the second isomer of tetra-*O*-galloyl-*β*-D-glucose (**45**) was detected only in the extracts obtained from unripe fruits. Mono-, tri-, and tetra-*O*-galloyl-*β*-D-glucose have been previously detected in *C. mas* stones [27] and flesh [16].

The second group of tannins present in the *C. mas* extracts was ellagitannins. In the examined extracts, we identified monomeric (**8**), dimeric (**9**, **14**, **21**, **26**, **28**, **29**, **34**–**36**, **39**, **49**), and trimeric ellagitannins (**16**, **18**, **22**–**24**, **30**–**32**, **38**, **41**, **48**). The identified ellagitannins consist of a glucose core with galloyl and hexahydroxydiphenoyl (HHDP) groups attached to the glucose. We identified 29 ellagitannins, including gemin D (**8**), two bis-HHDP hexoside isomers (**9**, **14**), three camptothin A isomers (**10**, **12**, **17**), two tellimagrandin I isomers (**19**, **25**), tellimagrandin II (**40**), seven cornusiin A isomers (**21**, **26**, **28**, **29**, **35**, **39**, **49**), seven cornusiin C isomers (**24**, **30**–**32**, **38**, **41**, **48**), four cornusiin F isomers (**16**, **18**, **22**, **23**), and two compounds identified as either camptothin B or cornusiin D (**34**, **36**). The fragmentation patterns of these ellagitannins have been thoroughly described by Przybylska et al. [27]. Cornusiins A–G and camptothins (A and B) are characteristic compounds of Corni Fructus (*Cornus officinalis* fruits) [39,40,41]. They have also been identified in *C. mas* stones [27], in the flesh of both ripe and unripe *C. mas* fruits [16], and in resin-purified extracts obtained from the red cultivar ‘Podolski’ as well as the yellow cultivars ‘Flava’ and ‘Yantarnyi’ [26].

Compounds **15**, **33**, **37**, and **53** belong to the non-phenolic group of compounds known as iridoids. Compound 15 produced a pseudomolecular ion at *m*/*z* 375 and a fragment ion at *m*/*z* 213 and was identified as loganic acid. Compounds **33** and **37** displayed formic acid–associated ions at *m*/*z* 403 [M–H + HCOO]^−^ and 435 [M–H + HCOO]^−^, respectively, and were identified as sweroside and loganin. The association of loganin and sweroside with formic acid has been previously described by Kucharska and Fecka [42]. Compound **53** produced a pseudomolecular ion at *m*/*z* 541 and a fragment ion at *m*/*z* 169 and was identified as cornuside. All identified iridoids have been previously reported in *C. mas* fruits by Deng et al. [31].

### 2.3. Quantification of Phenolic Compounds and Iridoids in C. mas Extracts

The contents of biologically active compounds in *C. mas* extracts are summarized in Table 4. Not all compounds listed in Table 3 were quantified because the UV analytical signal was low for many of them, and co-elution hindered accurate quantification. The polyphenol and iridoid contents in the brine of selected lactofermented unripe fruits are presented in Table S1.

The total anthocyanin content was 4.85 mg/g in the extract. This value was lower than that in our previous study, in which the resin-purified extract from *C. mas* cultivar ‘Podolski’ contained 22.01 mg/g of dry extract [21]. Cyanidin-3-*O*-galactoside was the predominant anthocyanin in the ripe fruit extract, accounting for 56% of the total anthocyanins, with a concentration of 2.71 mg/g of extract.

The phenolic acid contents in the extracts from ripe, unripe, and fermented fruits were 5.50, 7.73, and 22.3 mg/g, respectively. The highest concentration of caftaric acid was observed in extracts from ripe fruits, whereas the highest concentration of coutaric acid was found in extracts from fermented fruits. Fermented fruit extracts contained four times more gallic acid than ripe fruit extracts and 2.8 times more than unripe fruit extracts. The increase in gallic acid content resulted from the hydrolysis of hydrolyzable tannins during fermentation, caused by the action of bacterial enzymes.

In the fermented fruit extract, we identified only one flavonol, quercetin-3-*O*-glucuronide, which was present at 1.11 ± 0.01 mg/g of the extract. In the unripe fruit extract, we also detected quercetin-3-*O*-rutinoside, quercetin-3-*O*-hexoside (galactoside or glucoside), and kaempferol-*O*-glucoside; however, these compounds were present only in trace amounts that did not allow quantification. The ripe fruit extract was characterized by the highest flavonol content (6.48 mg/g of extract). Previously, the total flavonol content in resin-purified extracts from the *C. mas* cultivar ‘Podolski’ was reported as 2.41 mg/g of dried extract [21], 5.93 mg/g [26], and 5.53 mg/g [34]. These differences may be due to the fruits being harvested in different years, as well as variations in extraction conditions.

The most abundant compound and the predominant iridoid in all examined extracts was loganic acid. The extract from ripe fruits contained the highest concentration of loganic acid (313 ± 8.11 mg/g of extract). The amounts of loganic acid, sweroside, and cornuside did not differ significantly between the extracts from unripe and fermented fruits.

The total content of hydrolyzable tannins selected for quantification was 1.6-fold higher in the extract from unripe fruits compared with the extract from fermented fruits (75.3 vs. 47.6 mg/g of extract). The amount of hydrolyzable tannins decreases both during ripening and during the fermentation process. The degradation of tannins during fermentation occurs through depolymerization by tannase (a gallate decarboxylase) produced, for example, by *Lactobacillus plantarum*, which hydrolyzes tannins into gallic acid and glucose [43]. The high content of gallic acid in the extract may be important for its potential use in supporting the treatment of diseases associated with oxidative stress or inflammation. Gallic acid is rapidly absorbed in the gastrointestinal tract after oral administration and is distributed via the circulatory system to organs such as the heart, kidneys, liver, lungs, spleen, and brain. Despite this, its bioavailability remains poor, and before any clinical application, it should be enhanced using nanotechnology-based approaches, for example, through nanocarriers such as nanoparticles, nanoliposomes, or polymer–drug conjugates [44].

### 2.4. Total Phenolic Content and Antioxidant Activity of C. mas Extracts

In *C. mas* extracts, the TPC was measured using the Folin–Ciocalteu method, and in vitro antioxidant activity was evaluated using the ABTS, DPPH, and FRAP assays (Table 5). The TPC in the extract from unripe fruits was 1.4-fold higher than in the extract from fermented fruits and 2-fold higher than in the extract from ripe fruits. These results are consistent with those of Przybylska et al. [16], who reported that the amount of secondary metabolites in *C. mas* fruits decreases during ripening.

In both the DPPH and ABTS assays, unripe fruit extracts exhibited the highest antiradical activity, followed by fermented and ripe fruit extracts. Similarly, in the FRAP assay, the ferric-reducing power was greatest in unripe fruit extracts and lower in fermented and ripe fruit extracts. The antioxidant activity of the investigated extracts was elevated, as they were obtained from fruit juice that had been depectinized, purified from sugars and organic acids using ion-exchange resin, and subsequently lyophilized. For comparison, the antioxidant activity of fresh fruits from different cornelian cherry cultivars was 5.53–6.25 mmol Trolox (Tx)/kg fresh weight (FW) in the ABTS assay, 2.44–2.81 mmol Tx/kg FW in the DPPH assay, and 3.31–4.89 mmol Tx/kg FW in the FRAP assay [45].

### 2.5. α-Amylase and α-Glucosidase Inhibitory Activity of the C. mas Extracts

The *α*-amylase and *α*-glucosidase inhibitory activities did not differ significantly among the tested extracts, and all were less effective than the reference substance, acarbose. In our study, the IC_50_ values of acarbose were 0.05 mg/mL for *α*-amylase and 0.10 mg/mL for *α*-glucosidase. These findings contrast with those of Szczepaniak et al. [46], who reported that *C. mas* fruit extracts inhibited *α*-glucosidase more strongly than acarbose. In their study, the IC_50_ values for three *C. mas* cultivars ranged from 171 to 175 μg/mL, compared with 1.22 mg/mL for acarbose. Paun et al. [47] evaluated the α-amylase and *α*-glucosidase inhibitory activity of *C. mas* extracts obtained by different extraction methods. The IC_50_ values for *α*-amylase ranged from 0.11 ± 0.01 to 97.0 ± 2.70 μg/mL, compared to 8.12 ± 0.60 μg/mL for acarbose. For *α*-glucosidase, the IC_50_ values ranged from 98.2 ± 4.70 to 135 ± 6.20 μg/mL, while acarbose showed 168.4 ± 6.90 μg/mL. In the present study, the *α*-amylase and *α*-glucosidase inhibitory activity was less pronounced than that reported in the studies discussed above.

The ability to inhibit *α*-glucosidase and *α*-amylase has also been confirmed for *Prunus domestica* fruits. The inhibitory activity of extracts from 43 *P. domestica* cultivars toward *α*-amylase and α-glucosidase, expressed as IC_50_ values, ranged from 2.63 to 61.5 mg/mL and from 0.19 to 24.1 mg/mL, respectively. The ability to inhibit both enzymes was strongly associated with the abundance of flavan-3-ols and organic acids in *P. domestica* fruits [48]. These enzymes were also strongly inhibited by anthocyanin-rich bilberry (*Vaccinium myrtillus* L.) extract [49] and araçá (*Psidium cattleianum* Sabine) fruit extracts [50]. The IC_50_ values for bilberry extracts were 4.06 ± 0.12 mg/mL for *α*-amylase and 0.31 ± 0.02 mg/mL for *α*-glucosidase [49]. Purified extracts from two red araçá cultivars inhibited these enzymes more strongly than *P. domestica*, *V. myrtillus*, and the extracts examined in the present study. The IC_50_ values for *α*-amylase ranged from 870 to 21 µg/mL, and for *α*-glucosidase from 32 to 6 µg/mL [50]. Compared with other extracts, *C. mas* extracts appear to be a promising agent for inhibiting enzymes responsible for carbohydrate digestion. However, comparison of literature data on enzyme inhibition is limited by differences in fruit-extract preparation methods.

### 2.6. Effect of C. mas Extracts on Cellular Viability

**Figure 1:**
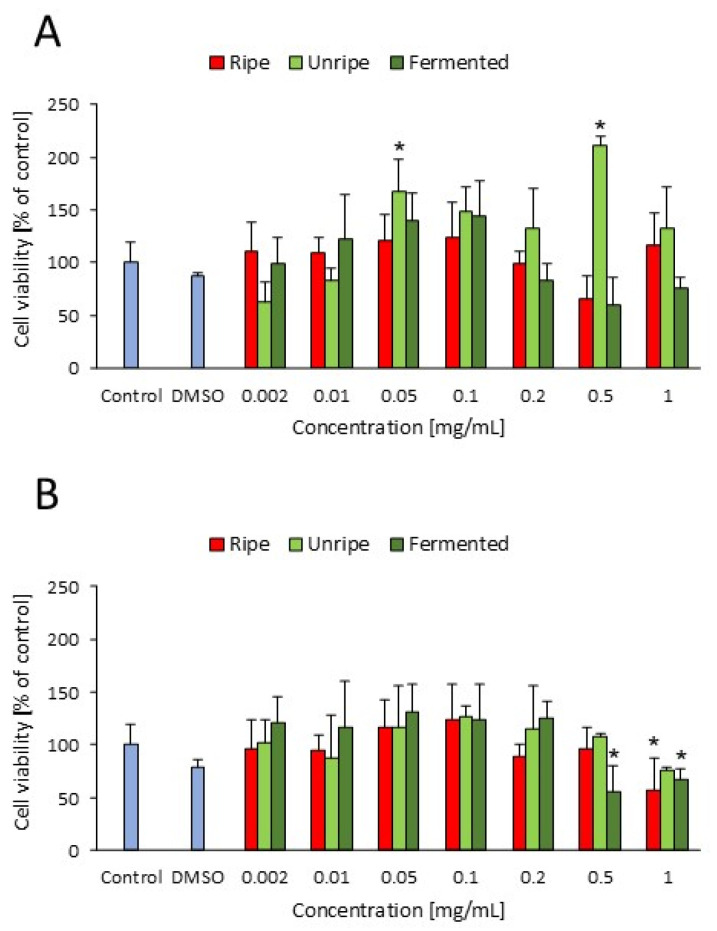
The cell viability measured using MTT test for ripe, unripe, and fermented fruit extracts after 24 h (**A**) and 48 h (**B**). * *p* < 0.05 compared to the control; ANOVA, Duncan’s test. Data are presented as mean ± SD.

Cell viability was evaluated after 24 and 48 h of treatment with fruit extracts (Figure 1) and brine from fermented fruits (Figure S9). After 24 h, the unripe fruit extract increased 3T3-L1 viability at concentrations of 0.05 and 0.5 mg/mL (*p* = 0.010 and *p* < 0.001, respectively), likely due to enhanced mitochondrial activity. An increase in cell viability was also observed in our previous study [21] after 24 h of treatment with ripe *C. mas* extract (cultivar ‘Podolski’) at concentrations of 0.05 and 0.2 mg/mL.

After 48 h, the ripe fruit extract decreased viability at 1 mg/mL (*p* = 0.010). Fermented fruit extract exerted the strongest effect, reducing viability at concentrations of 0.5 and 1 mg/mL (*p* = 0.006 and *p* = 0.023, respectively) (Figure 1). Based on the cell viability test, the concentrations selected for subsequent experiments were 0.05 mg/mL for the ripe fruit extract, 0.2 mg/mL for the unripe fruit extract, 0.1 mg/mL for the fermented fruit extract, and 5% for the brine.

### 2.7. Effect of C. mas Extracts on Glucose Uptake and Expression of Insulin-Related Genes

**Figure 2:**
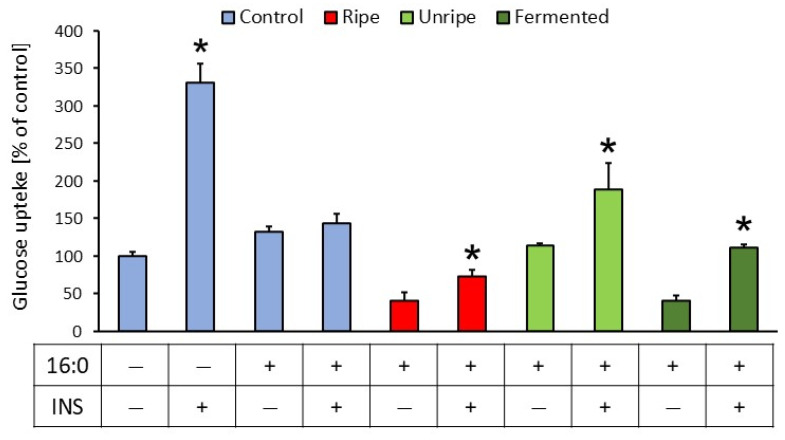
Glucose uptake in insulin-sensitive control adipocytes and insulin-resistant adipocytes (control and those treated with ripe fruit extract at 0.05 mg/mL, unripe fruit extract at 0.2 mg/mL, or fermented fruit extract at 0.1 mg/mL). Insulin resistance was induced with palmitic acid (16:0) at a concentration of 0.5 mM, and glucose uptake was assessed in the absence or presence of insulin stimulation (1 µM). * *p* < 0.05 compared to the baseline (INS−) of the corresponding variant; ANOVA, Duncan’s test. Data are presented as mean ± SD.

3T3-L1 fibroblasts were differentiated into adipocytes prior to glucose uptake assessment. Insulin resistance was induced with palmitic acid (16:0) at a concentration of 0.5 mM. Glucose uptake was evaluated in adipocytes with or without insulin stimulation (1 µL for 15 min). The ability to absorb glucose was measured in insulin-sensitive controls, insulin-resistant controls, and insulin-resistant adipocytes treated with extracts at concentrations determined by the MTT (3-(4,5-dimethylthiazol-2-yl)-2,5-diphenyltetrazolium bromide) assay (Figure 2). Glucose uptake was also assessed in insulin-resistant adipocytes treated with brine from unripe *C. mas* (Figure S10).

In insulin-sensitive adipocytes, glucose uptake after insulin stimulation was higher than that in non-stimulated cells (*p* < 0.001). In contrast, no significant difference in glucose uptake was observed between insulin-resistant cells with or without insulin stimulation (*p* = 0.546), confirming the successful induction of insulin resistance. Treatment of insulin-resistant adipocytes with extracts from ripe, unripe, and fermented *C. mas* fruits significantly enhanced glucose uptake following insulin stimulation compared to non-stimulated cells (*p* = 0.001, 0.003, and < 0.001, respectively) (Figure 2). Similarly, treatment with brine from fermented *C. mas* fruits increased glucose uptake in insulin-stimulated adipocytes compared to non-stimulated cells (*p* = 0.010) (Figure S10).

We previously reported that resin-purified extracts from ripe *C. mas* (‘Podolski’) significantly increased glucose uptake in insulin-resistant 3T3-L1 cells after insulin stimulation compared with cells not treated with insulin (*p* = 0.002). A less pronounced but still significant increase in insulin-stimulated glucose uptake after treatment with ripe *C. mas* ‘Podolski’ extract was observed in cells derived from human subcutaneous adipose tissue (SAT). The extract also exhibited a beneficial effect on glucose uptake in insulin-resistant cells derived from human visceral adipose tissue (VAT), but only when compared with insulin-resistant controls. Similarly, the resin-purified extract from a mixture of yellow *C. mas* cultivars (‘Yantarnyi’ and ‘Flava’) increased glucose uptake after insulin stimulation in insulin-resistant 3T3-L1 adipocytes (*p* = 0.046) [21].

In the available literature, relatively few studies have addressed the effects of fruit extracts on glucose uptake in adipocytes. Most research has primarily focused on lipid metabolism and the regulation of adipocyte differentiation [51]. A favorable effect on glucose uptake in adipocytes was demonstrated following treatment of 3T3-L1 cells with elderberry (*Sambucus nigra* L.) extract [52]. A beneficial effect on glucose uptake has also been observed following treatment with pure polyphenols, including gallic acid [53], flavonol glycosides [54], and ellagitannin metabolites [55].

To evaluate the possible mechanism underlying the improvement in insulin sensitivity in insulin-resistant 3T3-L1 mature adipocytes, the expression of key insulin pathway genes, *INSR* and *SLC2A4*, was assessed after 48 h of treatment with selected *C. mas* extracts and brine derived from fermented fruits. In our study, no alterations in the expression of *INSR* and *SLC2A4* (GLUT4) were detected following the treatment of insulin-resistant cells with extracts from unripe and fermented *C. mas* fruits or with brine derived from fermented fruits. The effect of insulin-stimulated glucose uptake in insulin-resistant adipocytes may occur through the regulation of other genes, such as *SIRT1*, *IRS1*, or *PI3K*, or via transcription factors, including *PPARG* [21,54].

Małodobra-Mazur et al. [21] reported that the primary mechanism regulating insulin sensitivity in insulin-resistant 3T3-L1 adipocytes following administration of *C. mas* extracts from red cultivars is the increased expression of *PPARG* compared to the control (*p* = 0.001 and *p* = 0.009). An increase in *SLC2A4* expression was observed only after the administration of yellow *C. mas* extracts (*p* = 0.016). Additionally, *INSR* expression increased in SAT and VAT cells after treatment with both *C. mas* extracts.

It has also been demonstrated that pure bioactive compounds present in cornelian cherry extracts modulate glucose uptake in adipocytes. Lim et al. [54] reported that the flavonol glycosides rutin (5 µM) and quercetin-3-*O*-glucoside (5 and 10 µM) significantly enhanced insulin-stimulated glucose uptake in mature 3T3-L1 adipocytes compared to the control. The authors suggested that the improvement in glucose uptake was mediated via AMPK activation and/or the Akt-dependent insulin signaling pathway [54].

Gallic acid (10 and 20 µM) increased glucose uptake in mature, differentiated 3T3-L1 adipocytes, although to a lesser extent than the reference compound, rosiglitazone. The authors suggested that glucose uptake in 3T3-L1 adipocytes was improved via Glut4 translocation, but the expression of *PPARG* and *C*/*EBPα* in mature 3T3-L1 adipocytes was also observed. Moreover, an increase in insulin sensitivity following gallic acid treatment has been reported to occur through the activation of Akt rather than the AMPK signaling pathway [53].

Although it is well established that ellagitannins exert antidiabetic effects by inhibiting *α*-amylase and *α*-glucosidase, information regarding their impact on glucose metabolism in adipocytes remains limited. Tolmie et al. [55] investigated the effects of urolithins, metabolites formed as a result of microbial metabolism of ellagitannins. Urolithin A and B (0.1, 1, and 10 µM) significantly increased glucose uptake in 3T3-L1 adipocytes (*p* < 0.05) compared to the reference compound metformin (65 µM). A limitation of this study is that glucose uptake was not assessed following insulin stimulation, and the underlying molecular mechanisms were not addressed. The beneficial effects of urolithins A and B (10 µM) on glucose uptake were also confirmed in mouse myoblasts (C2C12) and human hepatoma cells (HepG2), in comparison with metformin [55].

Information on the impact of iridoids, one of the main biologically active compounds present in *C. mas* fruits, on glucose uptake is also limited. To the best of our knowledge, no studies have evaluated glucose uptake after the administration of pure loganic acid, sweroside, or cornuside in 3T3-L1 adipocytes.

The most important limitation of this study is that the extracts were applied directly to the cell culture without considering the processes of absorption, metabolism, and biotransformation [51]. Future studies on digestion and metabolism in rodent and human models are required to better understand the transformation of the biologically active compounds present in the extracts. Further studies are required to elucidate the molecular mechanisms underlying the impact of *C. mas* extracts on insulin sensitivity in adipocytes. It would also be of interest to determine whether the improvement in adipocyte insulin sensitivity is primarily attributable to the major biologically active compounds present in the extract, or whether this effect is augmented through the synergistic interactions of the compounds contained within the *C. mas* extracts.

## 3. Materials and Methods

### 3.1. Chemicals and Reference Standards

Acetonitrile, methanol, and hydrochloric acid were purchased from POCh (Gliwice, Poland); formic acid from Sigma Aldrich (St. Louis, MO, USA); and acetic acid, sodium carbonate, and sodium acetate from Chempur (Piekary Śląskie, Poland). The 2,2′-azinobis (3-ethylbenzthiazoline-6-sulfonic acid) (ABTS), potassium persulfate, 2,4,6-tri(2-pyridyl)-s-triazine (TPTZ), 1,1-diphenyl-2-picrylhydrazyl (DPPH), ferrous chloride (FeCl_3_), and 6-hydroxy-2,5,7,8-tetramethylchroman-2-carboxylic acid (Trolox) were from Sigma Chemicals Co. (Steinheim, Germany). The reference substances were obtained from Phyproof Reference Substances (PhytoLab, Vestenbergsgreuth, Germany) (loganic acid, sweroside, cornuside, and cyanidin 3-*O*-glucoside), Supelco (Bellefonte, PA, USA) (loganin), Extrasynthese (Genay, France) (gallic acid and *p*-coumaric acid), Cayman Chemical Company (Michigan, MI, USA) (*trans*-caftaric acid), and Merck (Darmstadt, Germany) (*trans*-coutaric acid).

### 3.2. Plant Material

**Figure 3:**
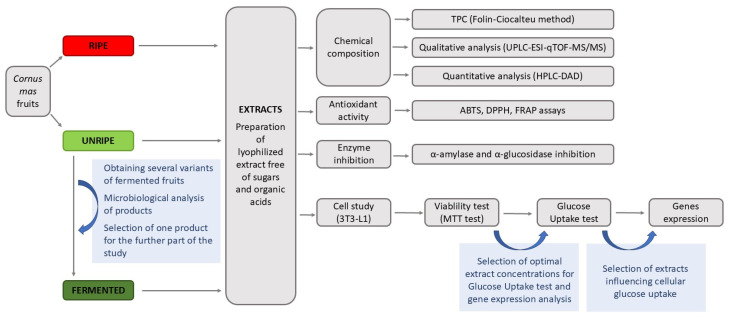
Study design. MTT, 3-(4,5-dimethylthiazol-2-yl)-2,5-diphenyltetrazolium bromide; TPC, total phenolic content.

The *C. mas* fruits, cultivar ‘Bolestraszycki,’ were harvested in 2023 in the Arboretum and Institute of Physiography in Bolestraszyce near Przemyśl, Poland. The voucher specimen (‘Bolestraszycki’ BDPA 3951) was deposited at the Herbarium of Arboretum in Bolestraszyce, Poland. The ripe fruits and a portion of unripe fruits were immediately frozen at −20 °C until extract preparation. The scheme of the experiments is shown in Figure 3.

### 3.3. Fermentation and Assessment of the Final Product

After rinsing, unripe *C. mas* fruits were fermented in 2% NaCl solution, prepared with commercially purchased thyme (1.3 g/L), pimento, bay leaf, starter strain, and a prebiotic (inulin at a concentration of 0.3%). The starter strain was previously isolated at the Institute of Fermentation Technology and Microbiology, Łódź, Poland, from lactofermented green tomatoes. Fermentation was carried out in single batches in jars at 12 °C for 3 months.

#### 3.3.1. Bacterial DNA Extraction, PCR Amplification and High-Throughput Sequencing

DNA from the bacterial strain was extracted using the GeneMATRIX Tissue & Bacterial DNA Purification Kit (EURx, Gdańsk, Poland), following the manufacturer’s protocol. The quality and quantity of the isolated DNA were assessed with a Qubit 2.0 Fluorometer (Invitrogen, Life Technologies, Carlsbad, CA, USA). Amplification of the 16S rRNA gene was performed using the universal primers 27f (5′-AGAGTTTGATCCTGGCTCAG-3′) and 1492r (5′-GGTTACCTTGTTACGACTT-3′). PCR was performed using REDTaq Ready Mix PCR Reaction Mix (Sigma-Aldrich) under the following conditions: initial denaturation at 94 °C for 2 min; 29 cycles of denaturation at 94 °C for 1 min, annealing at 49 °C, and extension at 72 °C for 3 min, followed by a final extension at 72 °C for 2 min. Sanger sequencing of the amplified 16S rRNA gene was performed by Genomed S.A. (Warsaw, Poland). The nucleotide sequence was analyzed using the BLAST+ 2.16.0 program (available at https://blast.ncbi.nlm.nih.gov/Blast.cgi (access date: 7 September 2025)) and then compared with the sequences available in the GenBank database (NCBI). The nucleotide sequence of the 16S rRNA gene of the *Levilactobacillus brevis* ZP HE1 strain was deposited in the GenBank database (NCBI) with accession number PX444865.

#### 3.3.2. Microbiological Analysis

Microbiological analyses were performed according to the ISO 6887 standard [56]. All microbiological media were obtained from Merck KGaA (Darmstadt, Germany). *Enterobacteriaceae* were enumerated on Violet Red Bile Dextrose agar (VRBD), total mesophilic counts (TMC) were determined on plate count agar (PCA), and yeasts and molds were enumerated on Dichloran Rose Bengal Chloramphenicol agar (DRBC). Lactic acid bacteria were enumerated on MRS broth supplemented with 2% agar and nystatin (AmBeed, Buffalo Grove, IL, USA). Plates were incubated at 30 °C for 48 h, except for the VRBD plates, which were incubated at 37 °C for 48 h.

#### 3.3.3. Analysis of Microbial By-Products by HPLC-PDA and HPLC-RI

Acetic, propionic, and lactic acids and ethanol were identified and quantified using a Finnigan Surveyor HPLC system (Thermo Fisher Scientific Inc., Waltham, MA, USA) equipped with a diode array detector (Surveyor-PDA Plus) and a refractive index detector (Surveyor-RI Plus). Short-chain fatty acids and lactic acid were quantified using a diode array detector, whereas ethanol was identified and quantified using a refractive index detector. Data acquisition was performed using ChromQuest 5.0 software (Thermo Fisher Scientific Inc., Waltham, MA, USA). Separation was performed on an Aminex HPX-87H column (300 × 7.8 mm; Bio-Rad, Hercules, CA, USA) at 60 °C, with a flow rate of 0.6 mL/min. The mobile phase consisted of 5 mmol/L sulfuric acid solution. Chromatograms were recorded at 210 nm with UV and RI detection.

### 3.4. Fruit Extracts Preparation

Frozen fruits with the addition of 1% citric acid solution (1% water solution) were heated in a Thermomix (Vorwerk, Wuppertal, Germany) for 3 min at 95 °C. The pulp was cooled, and a depectinizing agent (Pectinex Ultra Colour, Novozymes A/S, Bagsværd, Denmark) was added at a concentration of 1 g/1 kg of pulp. Depectinization was performed at 50 °C for 2 h. The pulp was then pressed using a hydraulic press (SRSE, Warsaw, Poland). The obtained juice was passed through an Amberlite XAD-16 resin column (Rohm and Haas, Chauny Cedex, France). Sugars and other polar compounds were removed using distilled water. Phenolic compounds were then eluted with 80% methanol. After methanol removal under reduced pressure at 40 °C (Buchi, Flawil, Switzerland), the extracts were freeze-dried (Alpha 1–4 LSC, Christ, Osterode am Harz, Germany).

### 3.5. Sample Preparation

For chemical analysis of total phenolic compounds, antioxidant activity, and *α*-amylase and *α*-glucosidase inhibition, 10 mg of extracts were dissolved in 5 mL of 80% methanol acidified with 1% HCl. Samples were diluted with distilled water (1:1 *v*/*v*) and filtered through a 0.45 µm PTFE filter (Chemland, Stargrad, Poland) for HPLC-PDA, Folin-Ciocalteau, and antioxidant analyses and a 0.22 µm PTFE filter (Chemland, Stargrad, Poland) for UPLC-ESI-qTOF-MS/MS analysis. For the cell study, 30 mg of the extracts were dissolved in DMSO and diluted in the appropriate Dulbecco’s Modified Eagle’s Medium (DMEM), according to the experiment (see Section 3.13, Section 3.14 and Section 3.15).

### 3.6. Identification of Phenolic Compounds by UPLC-ESI-qTOF-MS/MS

The identification of phenolic compounds was previously described by Przybylska et al. [27]. The compounds were identified using an Acquity ultra performance liquid chromatography (UPLC) system combined with a quadrupole-time of flight (Q-TOF) MS instrument (UPLC/Synapt Q-TOF MS, Waters Corp., Milford, MA, USA) in the negative and positive modes of electrical spray ionization (ESI). The compounds were identified based on fragmentation pathways and comparison with available literature data.

### 3.7. Quantification of Phenolic Compounds by HPLC-PDA

The HPLC-PDA analysis was performed according to Przybylska et al. [27] using a Dionex (Germering, Germany) system equipped with a diode array detector model Ultimate 3000. Data were collected using Chromeleon 7.2 software (Thermo Scientific Dionex, Sunnyvale, CA, USA). Separation was achieved using a Hypersil GOLD C18-column (250 mm × 4.6 mm; 5 μm) (Thermo Fisher Scientific Inc., Dartford, UK). HPLC chromatographs were recorded at specific wavelengths: 245 nm for iridoids, 254 nm for ellagic acid, 280 nm for flavan-3-ols and gallic acid, 320 nm for phenolic acids, 360 nm for flavonols, and 520 nm for anthocyanins. The quantity of phenolic compounds and iridoids was calculated using the linear regression equations of the external standards. Gallotannins and ellagitannins were identified based on MS data and quantified as gallic acid equivalents. The results are expressed as the mean of two replicates ± standard deviation and expressed as mg/g of extract.

### 3.8. Determination of Total Phenolic Content

Total phenolic content was determined by the Folin–Ciocalteu method described by Gao et al. [57], with slight modifications described by Przybylska et al. [16]. The absorbance was measured at a wavelength of 765 nm using a Synergy H1 multiplate reader (BioTek, Winooski, VT, USA). Each extract solution was prepared in two repetitions, and each solution was applied to the plate in four repetitions. A calibration curve was prepared for gallic acid. The results are expressed as the mean of two replicates ± standard deviation and expressed as g of gallic acid equivalent (GAE) per 100 g of extract.

### 3.9. Analysis of Antioxidant Capacity

The antioxidant activity was evaluated using the ABTS [58], DPPH [59], and FRAP [60] assays. All tests were performed according to modified protocols described by Przybylska et al. [16].

For all antioxidant assays, each extract solution was prepared in two replicates, and each solution was applied to the plate in four replicates. A calibration curve was prepared for Trolox. Results are presented as the mean of two replicates ± standard deviation and expressed as g of Trolox equivalent per g of freeze-dried extract. Measurements were taken using a microplate reader (Synergy H1; BioTek, Winooski, VT, USA).

### 3.10. α-Amylase Inhibition Assay

*α*-Amylase inhibition was determined according to the procedure described by Podsędek [61]. The absorbance was measured at 600 nm using a Synergy H1 multiplate reader (BioTek, Winooski, VT, USA). The inhibitory effect of *α*-amylase was calculated as follows:

*α*-amylase inhibition activity (%) = 100 × [1 − (AB − AA)/(AD − AC)] (1)

where AA is the absorbance of the reaction mixture with starch and fruit extract with amylase, and AB is the absorbance of the reaction mixture with starch and fruit extract without amylase. AC and AD were the absorbances of the reaction mixture containing starch and amylase or only starch, respectively. The extract solutions were prepared in two independent repetitions, and each repetition was measured in four replicates. The results are expressed as the mean ± SD of the two repetitions.

### 3.11. α-Glucosidase Inhibition Assay

*α*-Glucosidase inhibition was determined according to the procedure described by Podsędek [61]. After 20 min of incubation at 37 °C, 100 μL of 0.1 M sodium carbonate was added, and the absorbance was measured at a wavelength of 405 nm using a Synergy H1 multiplate reader (BioTek, Winooski, VT, USA). The inhibitory effect of *α*-glucosidase was calculated as follows:

*α*-glucosidase inhibition activity (%) = 100 × [1 − (AA − AB)/(AC − AD)] (2)

where AA is the absorbance of the sample, AB is the absorbance of the blank sample, consisting of the fruit extract, substrate, and enzyme added after incubation and stopping of the reaction with sodium carbonate solution, AC is the control, consisting of substrate, buffer, and enzyme, and AD is the blank control consisting of substrate, buffer, and enzyme added after incubation and stopping of the reaction with sodium carbonate solution. The IC_50_ value (in Section 3.10 and Section 3.11) was determined by linear regression analysis of the inhibition–concentration curve. Calculations were performed using data obtained for three extract concentrations. The extract solutions were prepared in two independent repetitions, and each repetition was measured in four replicates. The results are expressed as the mean ± SD of the two repetitions.

### 3.12. Cell Culturing and Adipocyte Differentiation

Mouse fibroblasts 3T3-L1 (ATCC, Manassas, VA, USA, CL-173™) were cultured in DMEM containing 4.5 g/L of glucose, L-glutamine, and sodium pyruvate (Corning, Manassas, VA, USA) supplemented with 10% Fetal Calf Serum (FCS, Sigma Aldrich, St. Louis, MO, USA) and an antibiotic mix including 50 U/mL penicillin and 50 µg/mL streptomycin (Corning). Cells were cultured in a humidified incubator with 5% CO_2_ at 37 °C. The cells were seeded on plates, and on the day of 100% confluence, the medium was changed to differentiated medium, containing DMEM enriched with 10% fetal bovine serum (FBS, Sigma Aldrich), an antibiotic mix (Corning), 390 ng/mL dexamethasone, 115 µg/mL 3-isobutyl-1-methylxanthine, and 10 µg/mL insulin. After 3 days, the medium was replaced with a medium sustaining differentiation, containing DMEM, 10% FBS, antibiotic mix, and 10 µg/mL insulin. 3T3-L1 fibroblasts reached maturity two weeks after the initiation of differentiation.

### 3.13. Cell Viability

Prior to the experiments, a viability test was performed using 3-(4,5-dimethylthiazol-2-yl)-2,5-diphenyltetrazolium bromide (MTT). 3T3-L1 fibroblasts were seeded on the plates at a density of 3000 cells per well. On the following day, fruit extracts at concentrations ranging from 0.002 to 1 mg/mL were added to the plates. After 24 and 48 h of incubation, 20 µL of 5 mg/mL MTT stock was added to each well, resulting in a final MTT concentration of 833 µg/mL. Yellow MTT is reduced to purple formazan in the mitochondria of living cells. Following 3.5 h of incubation at 37 °C, the medium with MTT was removed. Purple formazan crystals were dissolved in DMSO (100 µL/well) by gentle shaking for 15 min. The absorbance was measured at a wavelength of 590 nm with a reference filter of 620 nm. Viability was determined in three independent experiments. In each experiment, the extract solutions were prepared once, and the measurement was performed in three replicates (three wells).

### 3.14. Insulin Resistance Induction and Glucose Uptake Test

Insulin resistance was induced by supplementing the medium with 0.5 mM palmitic acid (Sigma-Aldrich, St. Louis, MO, USA) for 48 h. Subsequently, the fruit extracts diluted in palmitic acid-supplemented medium (at a concentration chosen based on the viability test) were added to the plate for the next 48 h. The cells were then starved overnight in a medium without FBS. On the day of the experiment, half of the cells were stimulated with 1 µM insulin (Sigma Aldrich) in PBS for 15 min at 37 °C. Subsequently, 1 mM 2-deoxyglucose and the remaining reagents included in the Glucose Uptake-Glo™ Assay kit (Promega, Madison, WI, USA) were applied according to the manufacturer’s technical manual. After 2.5 h of incubation, luminescence was measured using a Victor3 1420 Multilabel Plate Reader (Perkin Elmer, Waltham, MA, USA). The extract solutions were prepared in a single replicate, but the measurement of each variant (one extract with or without insulin stimulation) was performed in four replicates (four wells).

### 3.15. RNA Isolation and Gene Expression

Cells used for DNA isolation were cultured and differentiated in 6-well plates. Controls and extracts were applied to the plates in three replicates for each experimental variant. RNA isolation was performed separately for each replicate, and the isolated RNA samples were treated as independent replicates in the Real-Time PCR analysis. Total cellular RNA was isolated using TriReagent (Sigma-Aldrich, St. Louis, MO, USA). Briefly, 1 mL of TriReagent was added to the cell pellet. After 8 min of incubation on ice, 200 µL of chloroform (Sigma Aldrich) was added. The samples were shaken and centrifuged at 4 °C for 20 min. The aqueous phase was transferred to an Eppendorf tube, and RNA was precipitated using isopropanol (Sigma-Aldrich). Next, the samples were centrifuged at 4 °C for 15 min, RNA was washed with 70% ethanol (Chempur, Karlsruhe, Germany), dried, and dissolved in molecular biology-grade water. Reverse transcription was performed using a commercial kit (High-Capacity Reverse Transcription Kit, ThermoFisher, Waltham, MA, USA).

Prior to Real-Time PCR, primers were designed manually, and their efficiency was checked using a standard curve. Only primers with an efficiency of R^2^ > 0.95 were applied to the experiment. Gene expression was measured using Fast SYBR Green Master Mix (ThermoFisher). The primer sequences were previously described by [62]. Relative gene expression was normalized to *β*-actin (housekeeping gene) and calculated using the delta-delta Ct (ΔΔCt) model.

### 3.16. Statistical Analysis

Statistical analysis was performed using Statistica 14.0.0.15 (TIBCO Software Inc., Palo Alto, CA, USA). To evaluate the significant differences between groups, one-way ANOVA followed by Duncan’s test was applied. The results are presented as mean ± standard deviation.

### 3.17. Chemical Structures

The chemical structures were created using the ACD/ChemSketch (Freeware) 2021.2.1, Advanced Chemistry Development, Inc. (Toronto, ON, Canada).

## 4. Conclusions

This work represents the first comprehensive comparative analysis of the chemical composition, antioxidant activity, and in vitro antidiabetic properties of extracts derived from ripe, unripe, and unripe lactofermented cornelian cherry (*Cornus mas*) fruits.

The research confirmed that extracts from ripe, unripe, and fermented *C. mas* fruits constitute valuable sources of iridoids and polyphenols, including flavonoids (derivatives of quercetin and kaempferol), hydrolyzable tannins (gallotannins and ellagitannins), and phenolic acids. Extracts from ripe fruits contained anthocyanins (derivatives of pelargonidin and cyanidin) and were characterized by the highest levels of loganic acid, cornuside, and *trans*-caftaric acid. Unripe fruit extracts exhibited the highest content of hydrolyzable tannins and demonstrated the strongest antiradical activity in ABTS and DPPH assays, as well as the greatest ferric ion-reducing power in the FRAP test. During fermentation, complex compounds were transformed by microbial enzymes, resulting in a reduced tannin content and an increased concentration of gallic acid in the extract from fermented fruits.

All analyzed extracts exhibited inhibitory activity against the carbohydrate-hydrolyzing enzymes *α*-amylase and *α*-glucosidase. However, the extracts inhibited these enzymes to a considerably lesser extent than the reference compound, acarbose. Furthermore, all tested extracts improved insulin-stimulated glucose uptake in 3T3-L1 cells. Extracts from unripe and fermented fruits did not affect *INSR* or *SLC2A4* expression, suggesting that their antidiabetic activity may act through alternative molecular mechanisms or other molecular pathways. Based on these findings, extracts from unripe and fermented *C. mas* fruits may be promising agents for alleviating insulin resistance and preventing type 2 diabetes.

## Figures and Tables

**Table 1:** Microbiota of products from fermented unripe cornelian cherry fruits (log CFU/mL).

No.	Variant of Fermented Cornelian Cherry Fruits	*Enterobacteriaceae*	Yeasts	Molds	Lactic Acid Bacteria	Total Mesophilic Count
1	Cornelian cherry	2.98 ± 0.06	5.49 ± 0.10	<1	<1	5.58 ± 0.42
2	Cornelian cherry + *L. brevis* ZP HE1	2.00 ± 0.60	3.08 ± 0.36	1.48 ± 0.25	5.87 ± 0.61	5.90 ± 0.48
3	Cornelian cherry + thyme	<1	5.58 ± 0.03	<1	<1	5.38 ± 0.62
4	Cornelian cherry + thyme + inulin	<1	6.41 ± 0.74	<1	<1	6.43 ± 0.09
5	Cornelian cherry + thyme + inulin + *L. brevis* ZP HE1	<1	4.75 ± 0.34	<1	6.99 ± 0.70	4.81 ± 0.56

Data are presented as mean ± SD.

**Table 2:** Fermentation by-products in the brines of fermented unripe cornelian cherry fruits.

No.	Variant of Fermented Cornelian Cherry Fruits	Lactic Acid	Acetic Acid	Propionic Acid	Ethanol
		[mg/100 mL]	[mg/100 mL]	[mg/100 mL]	[%]
1	Cornelian cherry	132 ± 1.90	86.3 ± 0.50	295 ± 1.00	1.52 ± 0.20
2	Cornelian cherry + *L. brevis* ZP HE1	458 ± 6.00	95.2 ± 7.05	121 ± 0.63	1.30 ± 0.09
3	Cornelian cherry + thyme	155 ± 2.11	43.9 ± 0.50	99.0 ± 5.00	0.34 ± 0.05
4	Cornelian cherry + thyme + inulin	78.5 ± 2.03	83.1 ± 0.48	105 ± 0.60	1.21 ± 0.02
5	Cornelian cherry + thyme + inulin + *L. brevis* ZP HE1	519 ± 9.06	134 ± 2.00	84.4 ± 2.60	0.47 ± 0.10

Data are presented as mean ± SD.

**Table 3:** UPLC-ESI-qTOF-MS/MS analysis of *C. mas* fruit extracts.

No.	*t*_R_ (min)	MS1 (*m*/*z*)	MS2 Other Ions (*m*/*z*)	Assigned Identification	References	Fruit Extract
Positive ion mode, ESI^+^						
1	3.69	449	287	Cyanidin 3-*O*-galactoside	[25,26]	RF
2	3.93	595	287	Cyanidin 3-*O*-robinobioside	[25,26]	RF
3	4.19	433	271	Pelargonidin 3-*O*-galactoside	[25,26]	RF
4	4.43	579	271	Pelargonidin 3-*O*-robinobioside	[25,26]	RF
Negative ion mode, ESI^−^						
5	1.47	331	169, 125	Mono-O-galloyl-*β*-D-glucose	[27]	RF, UF, FF
6	1.61	169	125	Gallic acid	[28]	RF, UF, FF
7	1.86	361	169, 125	7-*O*-galloyl-D-sedoheptulose	[28,29,30]	RF, UF, FF
8	2.21	633	301, 275, 249, 169, 125	Gemin D	[27]	RF, UF, FF
9	2.66	783	481, 331, 301, 275, 169, 125	Bis-HDDP-hexoside (1)	[27]	RF, UF, FF
10	2.98	1417 708 [M − 2H]^−2^	1245, 1115, 785, 765, 633, 613, 450, 301, 275, 168, 125	Camptothin A (1)	[27]	RF, UF, FF
11	3.19	311	179, 149	*trans*-Caftaric acid	[26,28]	RF, UF, FF
12	3.40	1417 708 [M − 2H]^−2^	1247, 785, 765, 633, 613, 451, 301, 275, 169, 125	Camptothin A (2)	[27]	RF, UF, FF
13	3.43	341	211, 195, 163	*p*-Coumaric acid derivative	[26]	RF, UF, FF
14	3.61	783	633, 481, 301, 275, 249	Bis-HDDP-hexoside (2)	[27]	RF, UF, FF
15	3.78	375 751 [2M − H]^−^ 1127 [3M − H]^−^	213, 195, 169, 151, 125, 101	Loganic acid	[26,28]	RF, UF, FF
16	3.82	1100 [M − 2H]^−2^	1569, 1417, 1247, 785, 765, 633, 613, 483, 451, 313, 301, 275, 271, 249, 169, 125	Cornusiin F (1)	[27]	RF, UF, FF
17	3.89	1417 708 [M − 2H]^−2^	1247, 935, 785, 765, 633, 613, 451, 301, 275, 249, 169, 125	Campthothin A (3)	[27]	RF, UF, FF
18	4.06	2201 1100 [M − 2H]^−2^	1567, 1247, 785, 765, 633, 613, 483, 301, 169, 125	Cornusiin F (2)	[27]	RF, UF, FF
19	4.10	785	765, 633, 483, 451, 313, 301, 275, 249, 169, 125	Tellimagrandin I (1)	[27,28]	RF, UF, FF
20	4.29	635	313, 169, 125	Tri-*O*-galloyl-*β*-D-glucose	[27]	RF, UF, FF
21	4.34	1569 784 [M − 2H]^−2^	1417, 1247, 935, 785, 765, 633, 451, 301, 275, 249, 169, 125	Cornusiin A (1)	[27]	RF, UF, FF
22	4.40	1100 [M − 2H]^−2^	1569, 785, 765, 633, 613, 483, 451, 313, 301, 275, 271, 249, 169, 125	Cornusiin F (3)	[27]	RF, UF, FF
23	4.87	1100 [M − 2H]^−2^	1569, 1247, 933, 785, 765, 633, 613, 483, 451, 313, 301, 275, 271, 249, 169, 125	Cornusiin F (4)	[27]	RF, UF, FF
24	4.90	2353 1176 [M − 2H]^−2^	935, 785, 633, 451, 331, 301, 275, 249, 169, 125	Cornusiin C (1)	[27]	RF, UF, FF
25	4.90	785	765, 633, 483, 450, 313, 301, 275, 249, 168, 125	Tellimagrandin I (2)	[27,28]	RF, UF, FF
26	4.97	784 [M − 2H]^−2^	935, 785, 765, 633, 465, 451, 301, 275, 249, 169, 125	Cornusiin A (2)	[27]	RF, UF, FF
27	5.04	295	163, 149	*cis*-Coutaric acid	[26]	RF, UF, FF
28	5.22	1569 784 [M − 2H]^−2^	1249, 935, 785, 765, 633, 613, 483, 451, 331, 313, 301, 275, 249, 125	Cornusiin A (3)	[27]	RF, UF, FF
29	5.39	1569 784 [M − 2H]^−2^	1249, 935, 785, 765, 633, 613, 483, 451, 331, 313, 275, 249, 169, 125	Cornusiin A (4)	[27]	RF, UF, FF
30	5.46	2353 1176 [M − 2H]^−2^	1569, 1247, 933, 785, 765, 633, 451, 301, 275, 249, 169, 125	Cornusiin C (2)	[27]	RF, UF, FF
31	5.60	2353 1176 [M − 2H]^−2^	1569, 1249, 935, 785, 765, 633, 301, 275, 249, 169, 125	Cornusiin C (3)	[27]	RF, UF, FF
32	5.70	1176 [M − 2H]^−2^	1569, 785, 765, 633, 465, 451, 301, 275, 249, 169, 125	Cornusiin C (4)	[27]	RF, UF, FF
33	5.74	357 403 [M − H + HCOO]^−^	149, 195, 125, 101	Sweroside	[28,31]	RF, UF, FF
34	5.78	860 [M − 2H]^−2^	937, 935, 785, 633, 451, 301, 275, 249, 169, 125	Camptothin B or Cornusiin D (1)	[27]	RF, UF, FF
35	5.81	784 [M − 2H]^−2^	935, 785, 768, 633, 483, 451, 425, 331, 301, 275, 249, 169, 125	Cornusiin A (5)	[27]	RF, UF, FF
36	5.88	860 [M − 2H]^−2^	937, 785, 633, 451, 301, 275, 249, 169, 125	Camptothin B or Cornusiin D (2)	[27]	RF, UF, FF
37	5.95	389 435 [M − H + HCOO]^−^	227, 209, 197, 131, 101	Loganin	[28,31]	RF, UF, FF
38	6.12	1176 [M − 2H]^−2^	935, 785, 633, 451, 331, 301, 275, 249, 169, 125	Cornusiin C (5)	[27]	UF
39	6.16	1569 784 [M − 2H]^−2^	1417, 935, 785, 765, 633, 483, 451, 425, 331, 301, 275, 249, 169, 125	Cornusiin A (6)	[27]	RF, UF, FF
40	6.26	937	785, 465, 447, 313, 301, 275, 249, 169, 125	Tellimagrandin II	[27]	UF, FF
41	6.27	2353 1176 [M − 2H]^−2^	1569, 1417, 937, 785, 633, 613, 451, 301, 275, 249, 169, 125	Cornusiin C (6)	[27]	RF, UF
42	6.51	787	617, 465, 313, 169, 125	Tetra-*O*-galloyl-*β*-D-glucose (1)	[27]	RF, UF, FF
43	6.53	609	301	Quercetin 3-*O*-rutinoside	[32]	RF, UF
44	6.62	463	301	Quercetin 3-*O*-galactoside or glucoside	[32,33]	RF, UF
45	6.62	787	617, 313, 169, 125	Tetra-*O*-galloyl-*β*-D-glucose (2)	[27]	UF
46	6.76	301	275, 249	Ellagic acid	[27]	RF, UF, FF
47	6.76	477	301	Quercetin 3-*O*-glucuronide	[33,34]	RF, UF, FF
48	6.90	1176 [M − 2H]^−2^	1569, 935, 785, 633, 451, 301, 275, 249, 169, 125	Cornusiin C (7)	[27]	RF, UF, FF
49	7.14	1569 784 [M − 2H]^−2^	1417, 935, 785, 765, 633, 451, 425, 331, 301, 275, 249, 169, 125	Cornusiin A (7)	[27]	RF, UF
50	7.25	447	285	Kaempferol 3-*O*-glucoside	[28,32,33]	RF, UF
51	7.31	593	285	Kaempferol 3-*O*-rutinoside	[35]	RF
52	7.46	447	285	Kaempferol 3-*O*-galactoside	[32,35]	RF, UF
53	7.95	541	169	Cornuside	[25,31]	RF, UF, FF

*t*_R_, retention time; MS1, the first mass spectrum (pseudomolecular ion); MS2, the second mass spectrum (fragment ions); RF, ripe fruits; UF, unripe fruits; FF, fermented fruits.

**Table 4:** Content of polyphenols and iridoids in extracts from ripe, unripe, and fermented cornelian cherry fruits [mg/g of extract].

No.	Compound	Ripe Fruit Extract	Unripe Fruit Extract	Fermented Fruit Extract
**Anthocyanins**				
1	Cyanidin 3-*O*-galactoside	2.71 ± 0.02	n.a.	n.a.
2	Cyanidin 3-*O*-robinobioside	0.75 ± 0.01	n.a.	n.a.
3	Pelargonidin 3-*O*-galactoside	1.30 ± 0.01	n.a.	n.a.
4	Pelargonidin 3-*O*-robinobioside	0.09 ± 0.00	n.a.	n.a.
**Phenolic acids**				
6	Gallic acid	2.26 ± 0.05 ^b^	3.51 ± 0.14 ^b^	18.6 ± 0.82 ^a^
11	*trans*-Caftaric acid	1.07 ± 0.03 ^a^	0.73 ± 0.03 ^b^	0.37 ± 0.01 ^c^
13	*p*-Coumaric acid derivative	0.65 ± 0.01 ^c^	0.89 ± 0.03 ^b^	0.98 ± 0.03 ^a^
27	*cis*-Coutaric acid	1.30 ± 0.01 ^b^	1.22 ± 0.07 ^b^	1.50 ± 0.06 ^a^
46	Ellagic acid	0.22 ± 0.06 ^c^	0.98 ± 0.04 ^a^	0.85 ± 0.03 ^b^
**Flavonols**				
43	Quercetin 3-*O*-rutinoside	0.95 ± 0.02	t.a.	n.a.
44	Quercetin 3-*O* galactoside or glucoside	3.04 ± 0.01	t.a.	n.a.
47	Quercetin 3-*O*-glucuronide	1.10 ± 0.02 ^b^	2.37 ± 0.10 ^a^	1.11 ± 0.01 ^b^
50	Kaempferol 3-*O*-glucoside	0.10 ± 0.00	t.a.	n.a.
52	Kaempferol 3-*O*-galactoside	1.29 ± 0.00	n.a.	n.a.
**Iridoids**				
15	Loganic acid	313 ± 8.11 ^a^	190 ± 8.41 ^b^	211 ± 9.48 ^b^
33	Sweroside	15.7 ± 0.10 ^a^	12.1 ± 1.75 ^b^	12.6 ± 0.45 ^ab^
53	Cornuside	16.5 ± 0.27 ^a^	12.2 ± 0.55 ^b^	12.1 ± 0.49 ^b^
**Hydrolyzable tannins**				
5	Mono-*O*-galloyl-*β*-D-glucose	11.6 ± 0.25 ^b^	15.8 ± 0.74 ^a^	13.2 ± 0.68 ^b^
7	7-*O*-galloyl-D-sedoheptulose	8.79 ± 0.23 ^b^	12.4 ± 0.58 ^a^	9.76 ± 0.41 ^b^
10	Camptothin A (1)	2.46 ± 0.62 ^c^	8.26 ± 0.64 ^a^	4.35 ± 0.40 ^b^
12	Camptothin A (2)	2.04 ± 0.09 ^b^	6.55 ± 1.37 ^a^	3.53 ± 0.38 ^b^
21	Cornusiin A (1)	4.10 ± 0.40 ^b^	14.1 ± 1.69 ^a^	6.71 ± 0.39 ^b^
28	Cornusiin A (3)	5.83 ± 0.09 ^b^	18.2 ± 2.29 ^a^	10.0 ± 0.25 ^b^

Values are expressed as means ± standard deviation; small letters within the same rows are statistically different (*p* < 0.05, ANOVA, Duncan’s test); n.a., not abundant; t.a., trace amount.

**Table 5:** Total phenolic content, antioxidant activity, and enzyme inhibition of the analyzed extracts.

Extract	TPC (g GAE/100 g Extract)	ABTS	DPPH	FRAP	*α*-Amylase	*α*-Glucosidase
					Inhibition	
		(mmol Tx/100 g Extract)			(IC_50_ [mg/mL])	
Ripe fruit	25.9 ± 0.61 ^c^	222 ± 2.45 ^c^	191 ± 1.71 ^c^	163± 6.34 ^c^	1.95 ± 0.14	1.83 ± 0.12
Unripe fruit	51.4 ± 0.19 ^a^	351 ± 2.67 ^a^	291 ± 1.39 ^a^	251 ± 2.03 ^a^	1.71 ± 0.59	1.78 ± 0.02
Fermented fruit	37.8 ± 0.89 ^b^	333 ± 0.53 ^b^	261 ± 1.26 ^b^	225 ± 2.46 ^b^	1.88 ± 0.01	1.97 ± 0.08

Values are expressed as means ± standard deviation; small letters within the same column are statistically different (*p* < 0.05, ANOVA, Duncan’s test); GAE, gallic acid equivalent; TPC, total phenolic content; Tx, Trolox.

## Data Availability

Data is contained within the article or Supplementary Material.

## Supplementary Materials

The following supporting information can be downloaded at https://www.mdpi.com/article/10.3390/molecules30234625/s1, Figure S1: The chemical structures of gallotannins and ellagitannins in *Cornus mas* fruit extracts. Figure S2: The chemical structures of selected polyphenols and iridoids in *Cornus mas* fruit extracts. Figure S3: Total ion current (TIC) chromatogram obtained by UPLC-ESI-qTOF-MS/MS for the extract of unripe *Cornus mas* fruits. Figure S4: UPLC-PDA chromatogram of iridoids and ellagic (46) acid identified in extract from ripe *Cornus mas* fruits at *λ* = 254 nm. Figure S5: UPLC-PDA chromatogram of tannins and gallic acid (6) identified in extract from unripe *Cornus mas* fruits at *λ* = 280 nm. Figure S6: UPLC-PDA chromatogram of phenolic acids identified in extract from ripe *Cornus mas* fruits at *λ* = 320 nm. Figure S7: UPLC-PDA chromatogram of flavonols identified in extract from ripe *Cornus mas* fruits at *λ* = 360 nm. Figure S8: UPLC-PDA chromatogram of anthocyanins identified in extract from ripe *Cornus mas* fruits at λ = 520 nm. Figure S9: The viability test using MTT for brine from fermented *C. mas* fruits at various concentrations measured after 24 h (A) and 48 h (B); Figure S10: Glucose uptake in insulin-sensitive control adipocytes and insulin-resistant adipocytes (control and those treated with brine from fermented *C. mas* fruits at a concentration of 5%). Table S1: Content of polyphenols and iridoids in the brine of fermented *C. mas* products.

## Abbreviations

The following abbreviations are used in this manuscript:

IR	Insulin resistance
T2DM	Type 2 diabetes mellitus
TPC	Total phenolic content
LAB	Lactic acid bacteria
RF	Ripe fruit
UF	Unripe fruit
FF	Fermented fruit
SCOBY	Symbiotic culture of bacteria and yeast
HHDP	Hexahydroxydiphenoyl
MTT	3-(4,5-dimethylthiazol-2-yl)-2,5-diphenyltetrazolium bromide
SAT	Subcutaneous adipose tissue
VAT	Visceral adipose tissue
ABTS	2,2′-azinobis (3-ethylbenzthiazoline-6-sulfonic acid)
TPTZ	Potassium persulfate, 2,4,6-tri(2-pyridyl)-s-triazine
DPPH	2,2-diphenyl-1-picrylhydrazyl
VRBD	Violet Red Bile Dextrose
TMC	Total mesophilic counts
PCA	Plate count agar
DRBC	Dichloran Rose Bengal Chloramphenicol
FRAP	Ferric-reducing antioxidant power
