# Supplementary material for: Chemical Composition and In Vitro Antidiabetic Effect of Extracts from Ripe, Unripe, and Fermented Unripe Cornus mas L. Fruits
Source: Molecules. 2025 Dec 2;30(23):4625. doi: 10.3390/molecules30234625 (PMC12693535; doi:10.3390/molecules30234625)
Supplement: Supplementary file 1 [file molecules-30-04625-s001.zip › molecules-3988464-supplementary.pdf]

galloyl =

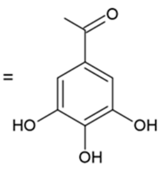

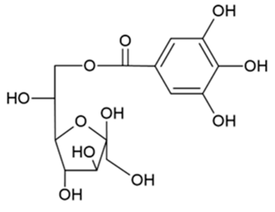

7-O-galloyl-D-sedoheptulose

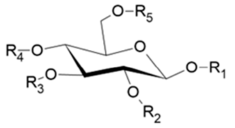

R_1=H, R_2=H, R_3=galloyl, R_4=H, R_5=H	Mono-*O*-galloyl-*β*-D-glucose
R_1=H, R_2=galloyl, R_3=galloyl, R_4=galloyl, R_5=H	Tri-*O*-galloyl-*β*-D-glucose
R_1=galloyl, R_2=galloyl, R_3=H, R_4=galloyl, R_5=galloyl	Tetra-*O*-galloyl-*β*-D-glucose

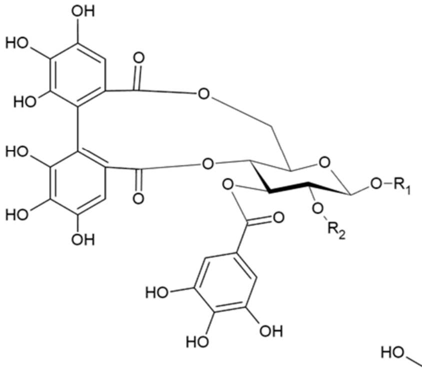

R_1=H, R_2=H	gemin D
R_1=H, R_2=galloyl	tellimagrandin I
R_1=galloyl, R_2=galloyl	tellimagrandin II

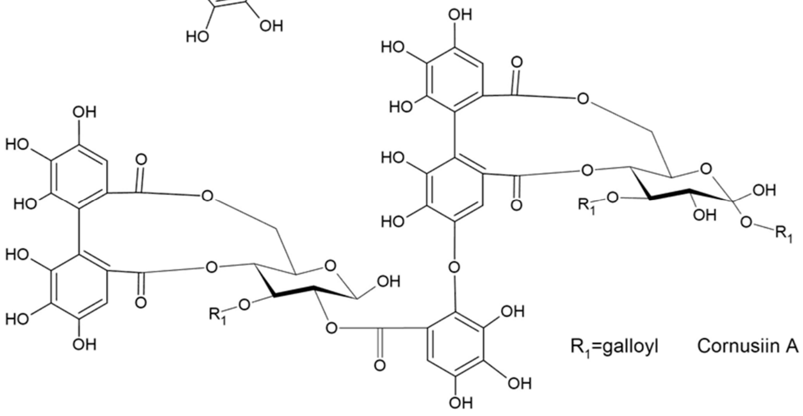

R_1=galloyl Cornusiin A

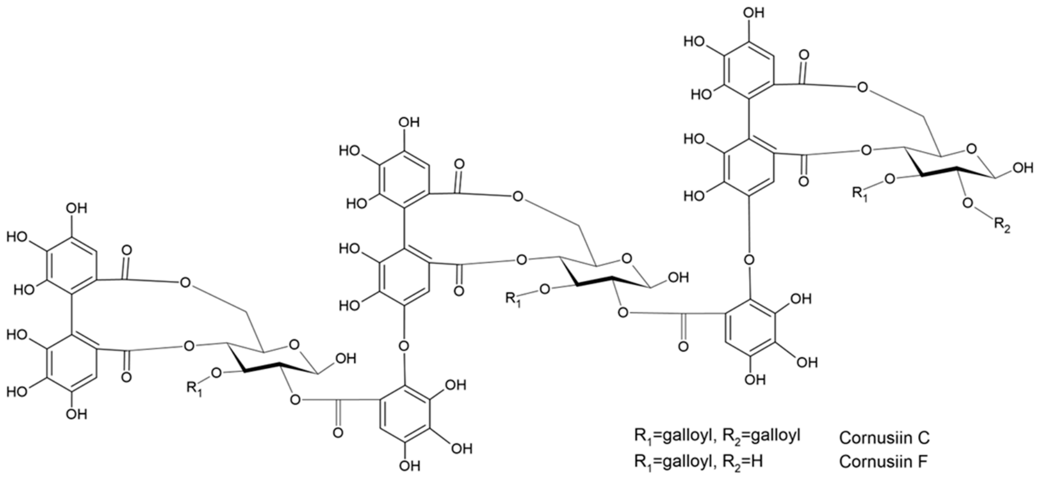

R_1=galloyl, R_2=galloyl	Cornusiin C
R_1=galloyl, R_2=H	Cornusiin F

Figure S1. The chemical structures of gallotannins and ellagitannins in *Cornus mas* fruit extracts.

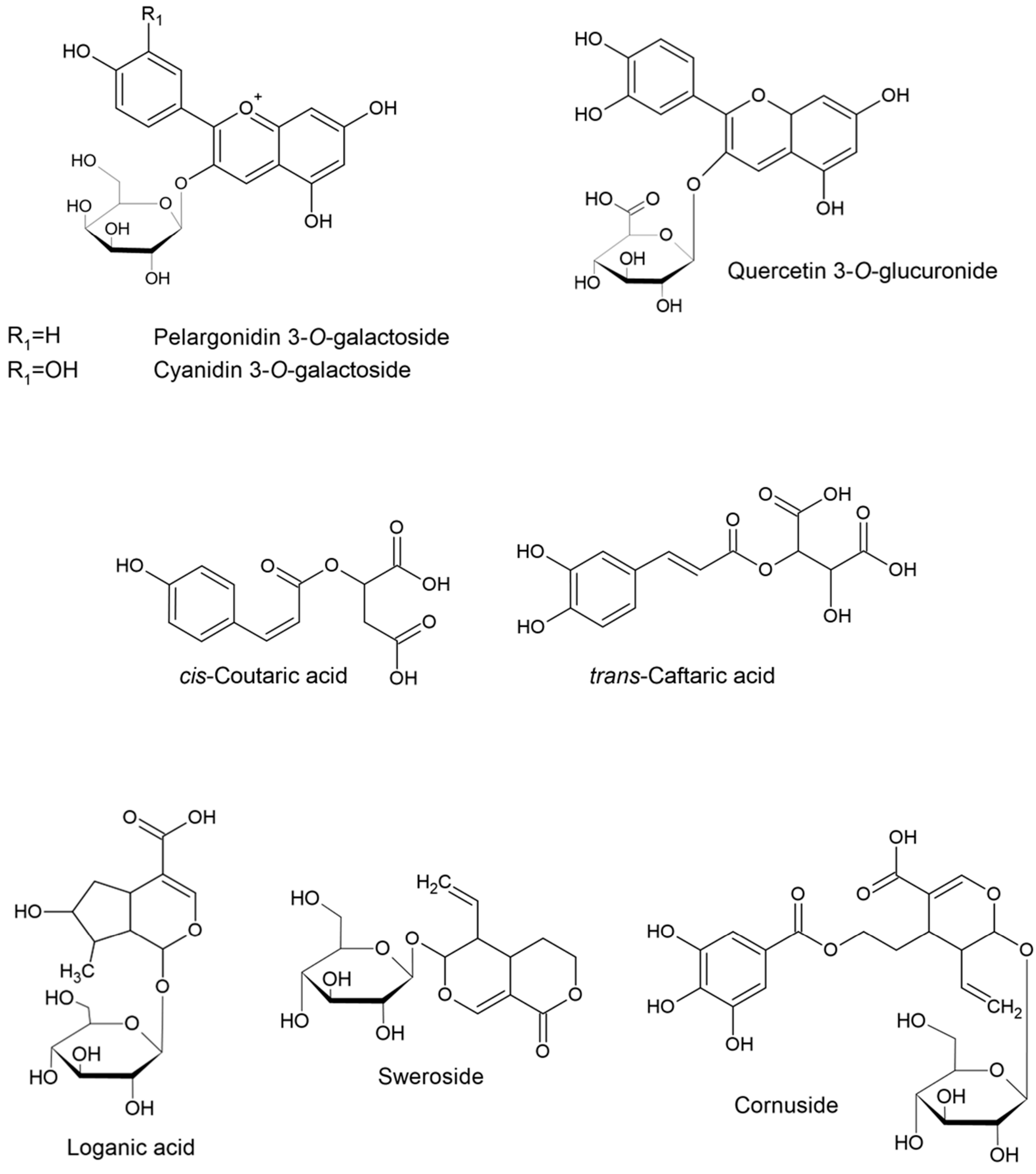

Figure S2. The chemical structures selected polyphenols and iridoids in *Cornus mas* fruit extracts.

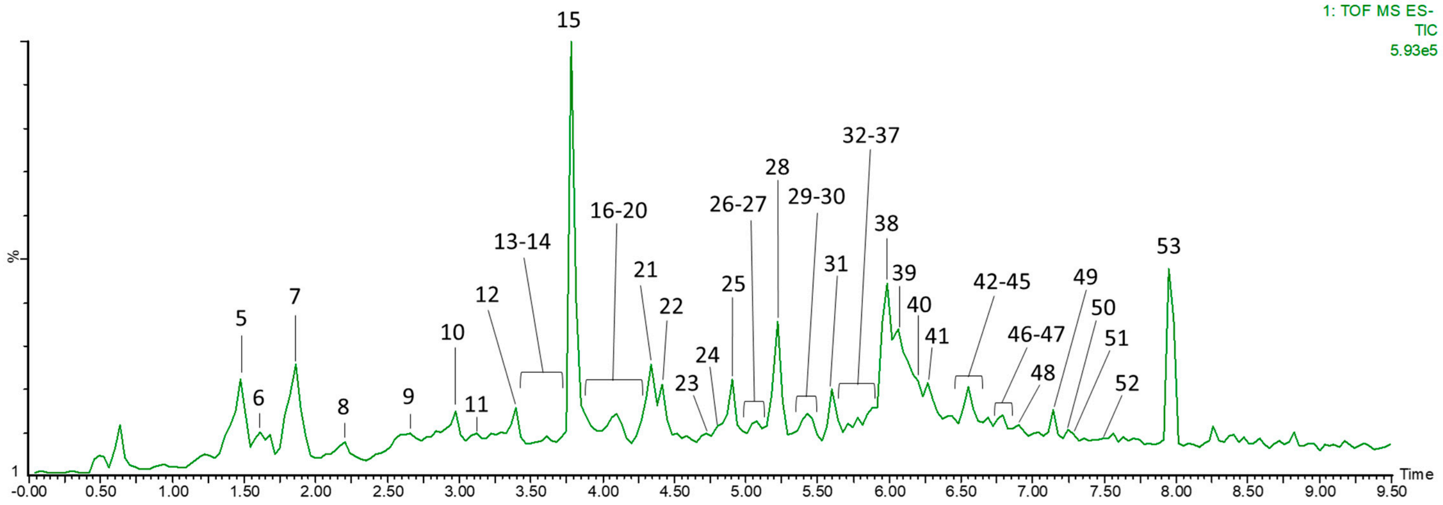

Figure S3. Total ion current (TIC) chromatogram obtained by UPLC-ESI-qTOF-MS/MS for the extract of unripe *Cornus mas* fruits. Peak numbers correspond to the compounds listed in Table 3.

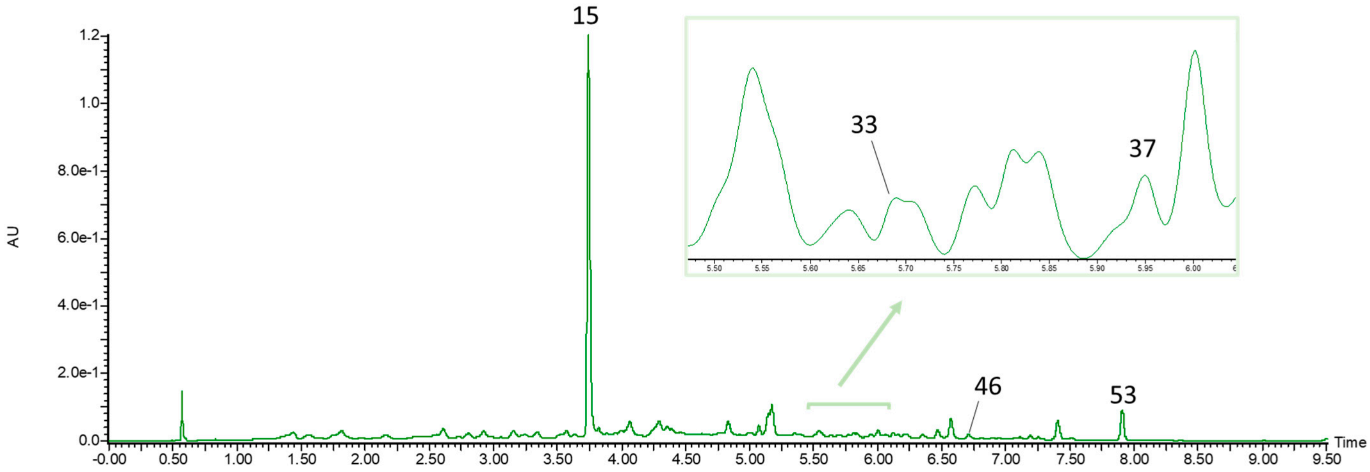

Figure S4. UPLC-PDA chromatogram of iridoids and ellagic (46) acid identified in extract from ripe *Cornus mas* fruits at λ = 254 nm. Peak numbers correspond to the compounds listed in Table 3.

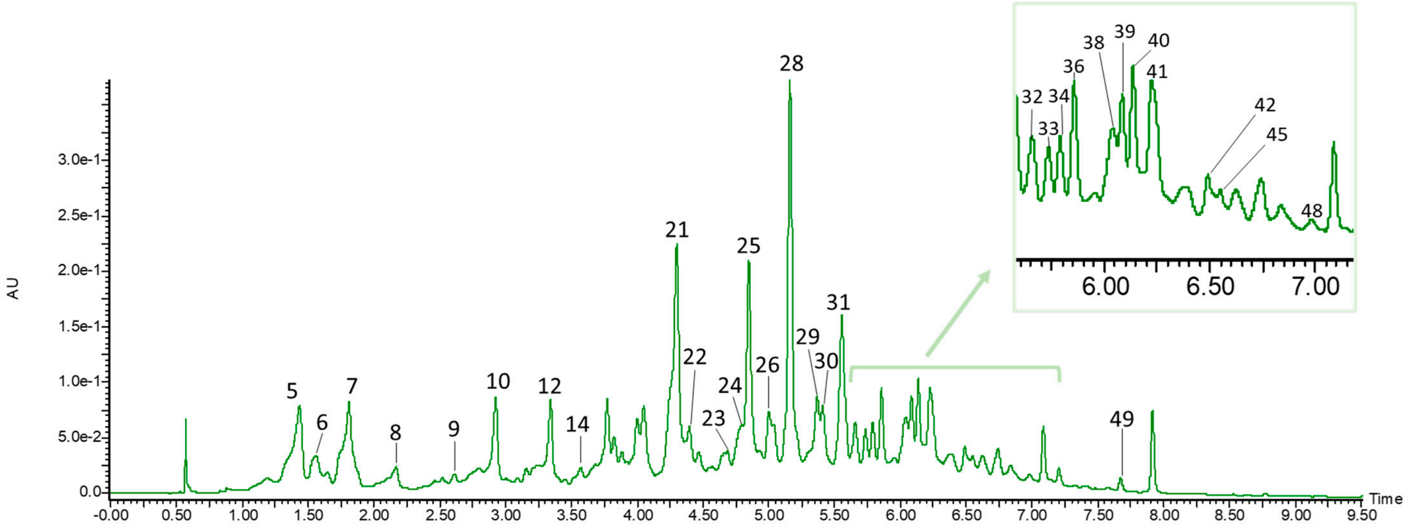

Figure S5. UPLC-PDA chromatogram of tannins and gallic acid (6) identified in extract from unripe *Cornus mas* fruits at λ = 280 nm. Peak numbers correspond to the compounds listed in Table 3.

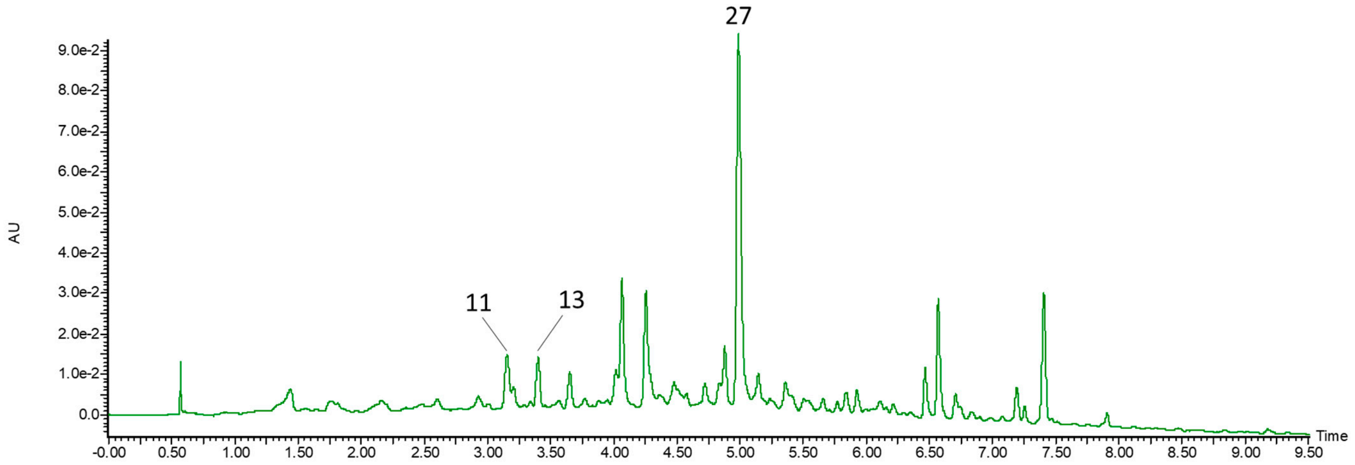

Figure S6. UPLC-PDA chromatogram of phenolic acids identified in extract from ripe *Cornus mas* fruits at λ = 320 nm. Peak numbers correspond to the compounds listed in Table 3.

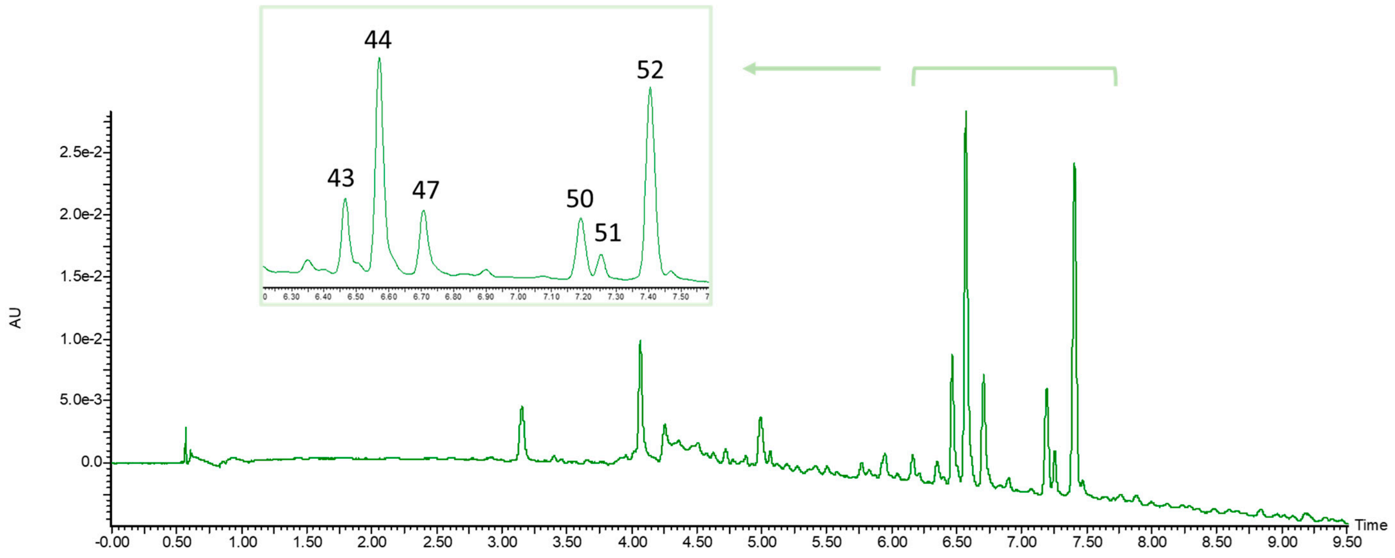

Figure S7. UPLC-PDA chromatogram of flavonols identified in extract from ripe *Cornus mas* fruits at λ = 360 nm. Peak numbers correspond to the compounds listed in Table 3.

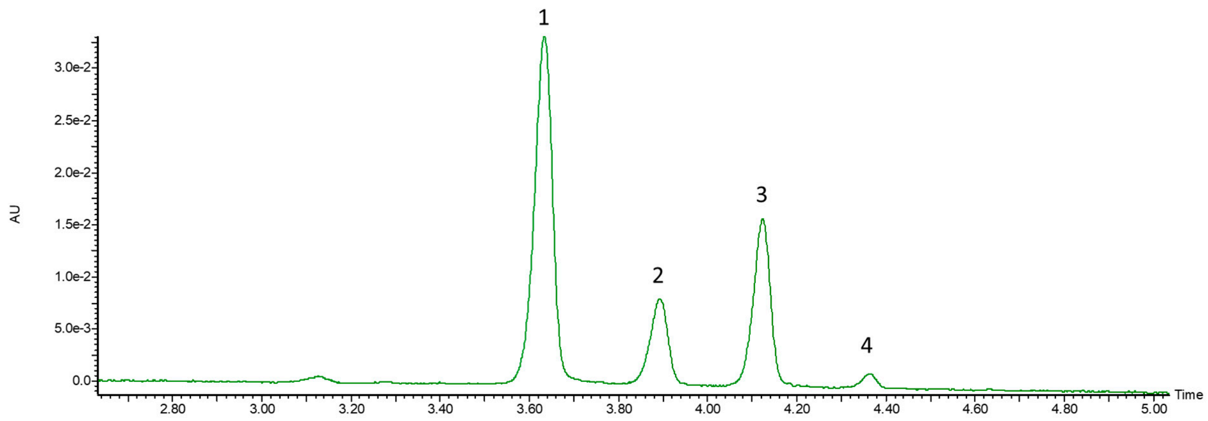

Figure S8. UPLC-PDA chromatogram of anthocyanins identified in extract from ripe *Cornus mas* fruits at λ = 520 nm. Peak numbers correspond to the compounds listed in Table 3.

Table S1. Content of polyphenols and iridoids in the brine of fermented *C. mas* products [mg/ 100 ml brine].

Compound	Brine
**Anthocyanins**	
Cyanidin 3-*O*-galactoside	n.a.
Cyanidin 3-*O*-robinobioside	n.a.
Pelargonidin 3-*O*-galactoside	n.a.
Pelargonidin 3-*O*-robinobioside	n.a.
**Phenolic acids**	
Gallic acid	10.2 ± 0.14
trans-Caftaric acid	0.23 ± 0.01
p-Coumaric acid derivative	0.28 ± 0.00
Coutaric acid	0.38 ± 0.01
Ellagic acid	0.21 ± 0.02
**Flavonols**	
Quercetin 3-*O*-rutinoside	n.a.
Quercetin 3-*O* galactoside or glucoside	n.a.
Quercetin 3-*O*-glucuronide	0.09 ± 0.01
Kaempferol *O*-hexoside	n.a.
Kaempferol 3-*O*-galactoside	n.a.
**Iridoids**	
Loganic acid	62.6 ± 0.86
Sweroside	1.36 ± 0.05
Cornuside	1.28 ± 0.05
**Hydrolyzable tannins**	
Mono-*O*-galloyl-*β*-D-glucose	3.67 ± 0.06
7-*O*-galloyl-D-sedoheptulose	3.42 ± 0.02
Camptothin A (isomer 1)	n.a.
Camptothin A (isomer 2)	n.a.
Cornusiin A (isomer 1)	n.a.
Cornusiin A (isomer 3)	n.a.

Values are expressed as means ± standard deviation; n.a., not abundant;

A

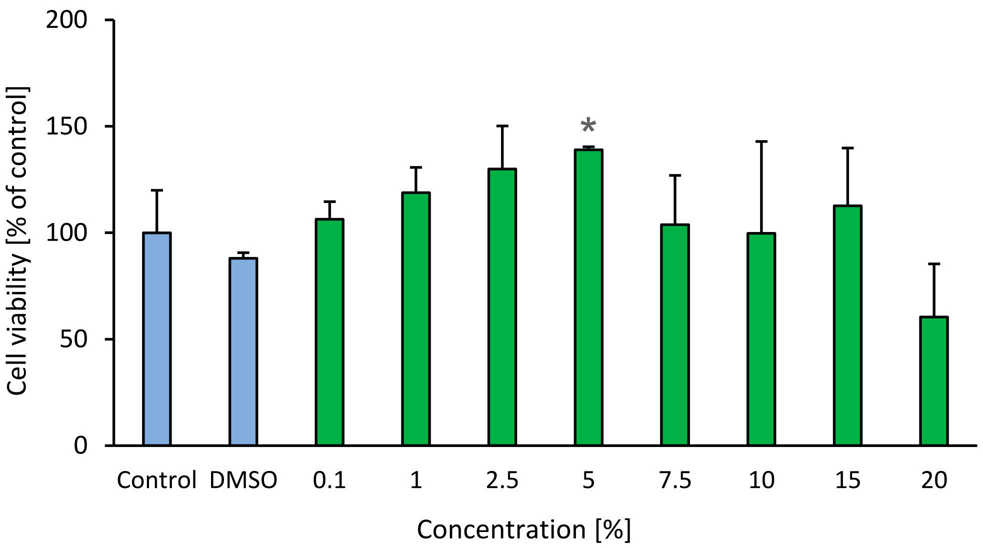

B

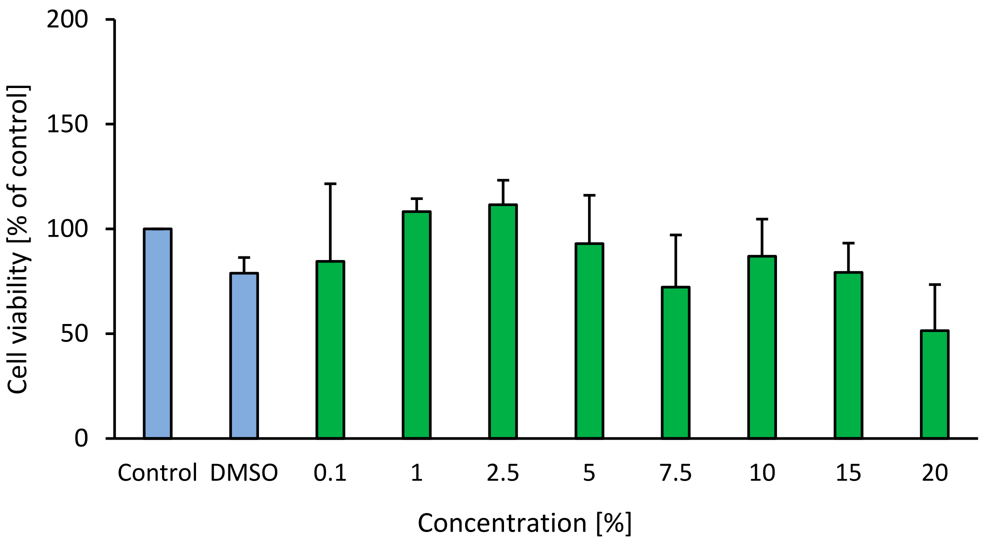

Figure S9. The viability test using MTT for brine from fermented *C. mas* fruits at various concentrations measured after 24 h (A) and 48 h (B). *$p < 0.05$ compared to the control; ANOVA, Duncan's test. Data are presented as mean ± SD.

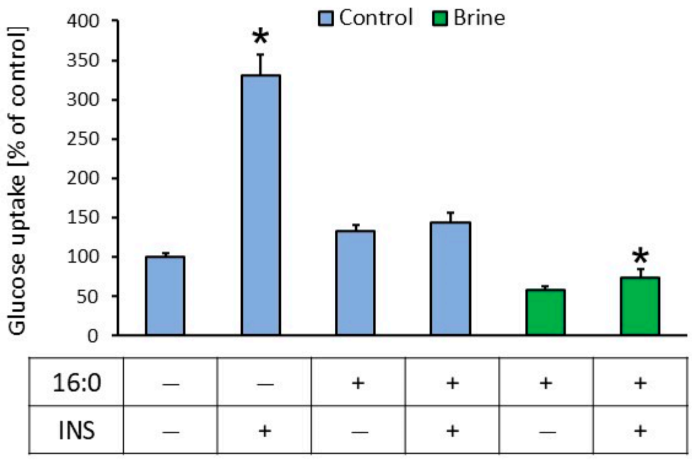

Figure S10. Glucose uptake in insulin-sensitive control adipocytes and insulin-resistant adipocytes (control and those treated with brine from fermented *C. mas* fruits at a concentration of 5%). Insulin resistance was induced with palmitic acid (16:0) at concentration 0.5 mM, and glucose uptake was assessed in the absence or presence of insulin stimulation (1 μM). * $p < 0.05$ compared to the baseline (INS −) of the corresponding variant; ANOVA, Duncan's test. Data are presented as mean ± SD.
